# Abnormal Prion Protein in Nasal Swab Specimens of Macaques Infected with Creutzfeldt-Jakob Disease

**DOI:** 10.3201/eid3112.250679

**Published:** 2025-12

**Authors:** Juraj Cervenak, Oksana Yakovleva, Cyrus Bett, Teresa Pilant, Kelly Rice, Lewis Shankle, David M. Asher, Luisa Gregori, Pedro Piccardo

**Affiliations:** US Food and Drug Administration, Center for Biologics Evaluation and Research, Silver Spring, Maryland, USA (J. Cervenak, O. Yakovleva, C. Bett, T. Pilant, K. Rice, L. Shankle, D.M. Asher, L. Gregori); Unidad Académica de Neuropediátria, Universidad de la República, Montevideo, Uruguay (P. Piccardo)

**Keywords:** Prions, nasal swabs, macaques, Creutzfeldt-Jakob disease, blood transfusion, RT-QuIC, PMCA, Uruguay

## Abstract

We transfused 4 macaques with blood of macaques previously infected with variant Creutzfeldt-Jakob disease, transmitting disease transmittal to 2 macaques (1 demonstrating clinical signs). Nasal swab specimens from both infected macaques became positive for disease-associated prion protein during the preclinical stage. Such samples are suitable for antemortem diagnosis during long incubation periods.

Variant Creutzfeldt-Jakob disease (vCJD) and sporadic CJD are among a group of rare, always fatal neurodegenerative disorders known as transmissible spongiform encephalopathies (TSEs) or prion diseases (*1*). Researchers have linked vCJD to human dietary exposure to the agent that caused bovine spongiform encephalopathy in cattle (*1*–*3*). The literature documents few transfusion-transmitted cases of vCJD in the early 2000s (*4*,*5*), but no new cases have been reported since 2007. The lack of recent transfusion-transmitted vCJD is reassuring, but a question lingers about the true prevalence of vCJD infections in the population and the iatrogenic risk they pose. This prevalence is difficult to estimate without a validated assay to identify infected persons during the preclinical phase of vCJD, which can last for many decades.

To achieve this goal, we developed a nonhuman primate model of vCJD to collect specimens (blood and nasal swabs) suitable to validate preclinical tests and to detect the presence of abnormal disease-associated prion protein (PrP^TSE^) in blood (*6*,*7*). We could not conduct studies with human samples because of the rarity of human TSEs and the impossibility of establishing the exact time of most human exposures to the infective agents. To validate the relevance of a macaque model, we attempted to mimic human transmissions of transfusion-transmitted vCJD by blood transfusion from infected to naive macaques and collected biologic samples for testing over a 10-year period.

## The Study

We transfused 4 uninfected macaques using blood from 3 macaques (CO7423, CO7422, and C16999) previously infected with vCJD (*6*) (Appendix). We collected 100-mL samples of blood from CO7423, at clinical onset and at terminal phase of illness, and immediately transfused the samples into 2 recipient macaques: CO1619 and 98CO19. Two years later, we transfused 2 macaques, DEIM and DFOO, with red blood cell–depleted blood prepared from whole blood of CO7422 and C16999 (Table 1). We euthanized CO1619 at 58 months posttransfusion (mpt) and euthanized 98CO19 at 104 mpt (8.7 years posttransfusion [ypt]) because of intercurrent illnesses (Table 1). DEIM developed early neurologic signs of mild ataxia and tremors at 104 mpt (8.7 ypt). Those symptoms slowly progressed to marked tremors, unsteadiness on the perch, unkept fur coat, and mild weight loss, all typical signs of vCJD in macaques. We euthanized DEIM 4 months after clinical onset. We euthanized DFOO at 120 mpt (10 ypt) when the macaque had reached the preselected experimental endpoint.

**Table 1:** Summary of data from study of abnormal prion protein in nasal swab specimens of macaques infected with Creutzfeldt-Jakob disease*

Donor macaque ID	Recipient macaque ID	Transfused sample	Clinical symptoms, ypt (mpt)	Survival time, ypt (mpt)	Age at endpoint, y	Reason for euthanasia
CO7423	CO1619	100 mL whole blood	NA	4.8 (58)	17	Unresectable pelvic tumor
CO7423	98CO19	100 mL whole blood	NA	8.7 (104)	23	Nonreducible inguinal hernia
CO7422	DEIM	Red blood cell–depleted 100 mL whole blood equivalent	8.7 (104)	9 (108)	12	Neurologic signs of vCJD
C16999	DFOO	Red blood cell–depleted 100 mL whole blood equivalent	NA	10 (120)	13	Planned end of study

*Inoculation of these animals was described previously (*6*). mpt, month posttransfusion; NA, not applicable; vCJD, variant Creutzfeld-Jakob disease; ypt, year posttransfusion.

We collected blood and nasal swab specimens during and at the end of the study. We harvested brains and other tissues from each macaque. We also collected cerebrospinal fluid from DEIM and DFOO. To detect PrP^TSE^, the biomarker of TSEs, we used 2 in vitro assays: real-time quaking-induced conversion (RT-QuIC) to assay nasal swab extracts, lymph nodes, and cerebrospinal fluid; and protein misfolding cyclic amplification (PMCA) to assay brain, spleen, and blood samples (*8*,*9*). All tests of tissues and fluids from CO1619 and DFOO were negative for PrP^TSE^, including neuropathological examinations to show spongiform degeneration and PrP^TSE^ deposits in formalin-fixed paraffin-embedded brain tissue (Table 2). We included samples from vCJD-infected animals and uninfected macaques in each test as controls.

**Table 2:** Summary of test results from study of abnormal prion protein in nasal swab specimens of macaques infected with Creutzfeldt-Jakob disease*

Recipient macaque ID	Clinical status	Tissue tested	Western blot†	RT-QuIC	PMCA	Histology	Total mice inoculated/ positive results‡
CO1619	Asymptomatic	Brain	Neg	Neg	Neg	Neg	40/0
		Spleen	ND	ND	Neg	ND	10/0
		Ileum	ND	ND	Neg	ND	15/0
		Blood	NA	NA	Neg	N/A	ND
		Nasal swab	ND	Neg	Neg	NA	ND
98CO19	Asymptomatic	Brain	Neg	Neg	Neg	Neg	51/0
		Blood	NA	ND	Neg	NA	ND
		Nasal swab	ND	W pos	Neg	NA	ND
		Lymph node	ND	W pos	Neg	Pos	ND
DEIM	Symptomatic	Brain	Pos	ND	ND	Pos	ND
		Blood	NA	NA	Pos	NA	ND
		Nasal swabs	ND	Pos	Pos	NA	ND
		CSF	ND	Pos	ND	NA	ND
DFOO	Asymptomatic	Brain	Neg	Neg	Neg	Neg	ND
		Blood	NA	NA	Neg	NA	ND
		Nasal swab	ND	Neg	Neg	NA	ND
		CSF	Neg	Neg	Neg	NA	ND

*CSF, cerebrospinal fluid; pos, TSE positive; W pos, weak TSE positive; neg, TSE negative; NA, not applicable; ND, not done; RT-QuIC, real-time quaking-induced conversion; TSE, transmissible spongiform encephalopathy. †We used 10% homogenates in phosphate-buffered saline, treated with 20 U/mL of benzonase nuclease for 30 minutes at room temperature with constant mixing, followed with 50 μg/mL proteinase K for 1 hour at 37°C to remove normal prion protein. Samples were processed for NuPAGE on 12% Bis-Tris precast gels (Thermo Fisher Scientific, https://www.thermofisher.com), prion protein detected using 3F4 mouse anti-PrP monoclonal antibody (Research Foundation for Mental Hygiene, New York State Institute for Basic Research; https://corporate.rfmh.org/). ‡We inoculated intracerebrally 30-µL aliquots of 1% tissue homogenates into transgenic mice overexpressing the bovine prion protein, TgBo110 (*10*). We monitored mice for 2 years for signs of vCJD and tested the brain of every mouse for PrP^TSE^ with real-time quaking-induced conversion.

**Figure 1:**
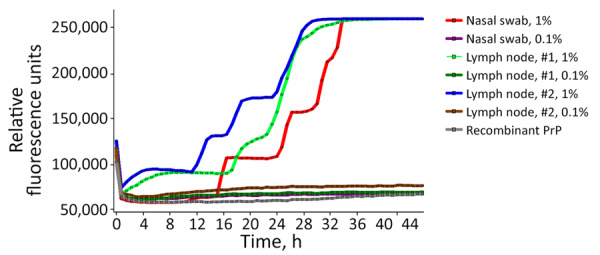
Results of real-time quaking-induced conversion testing of tissue samples from a study of macaques infected with Creutzfeldt-Jakob disease. Data based on nasal swab extracts and lymph nodes 1 and 2 from macaque 98CO19. Each line represents the change in fluorescent signal over time. We tested 1% and 0.1% homogenates; increase in fluorescent signal was observed only with 1% homogenates. PrP, prion protein.

**Figure 2:**
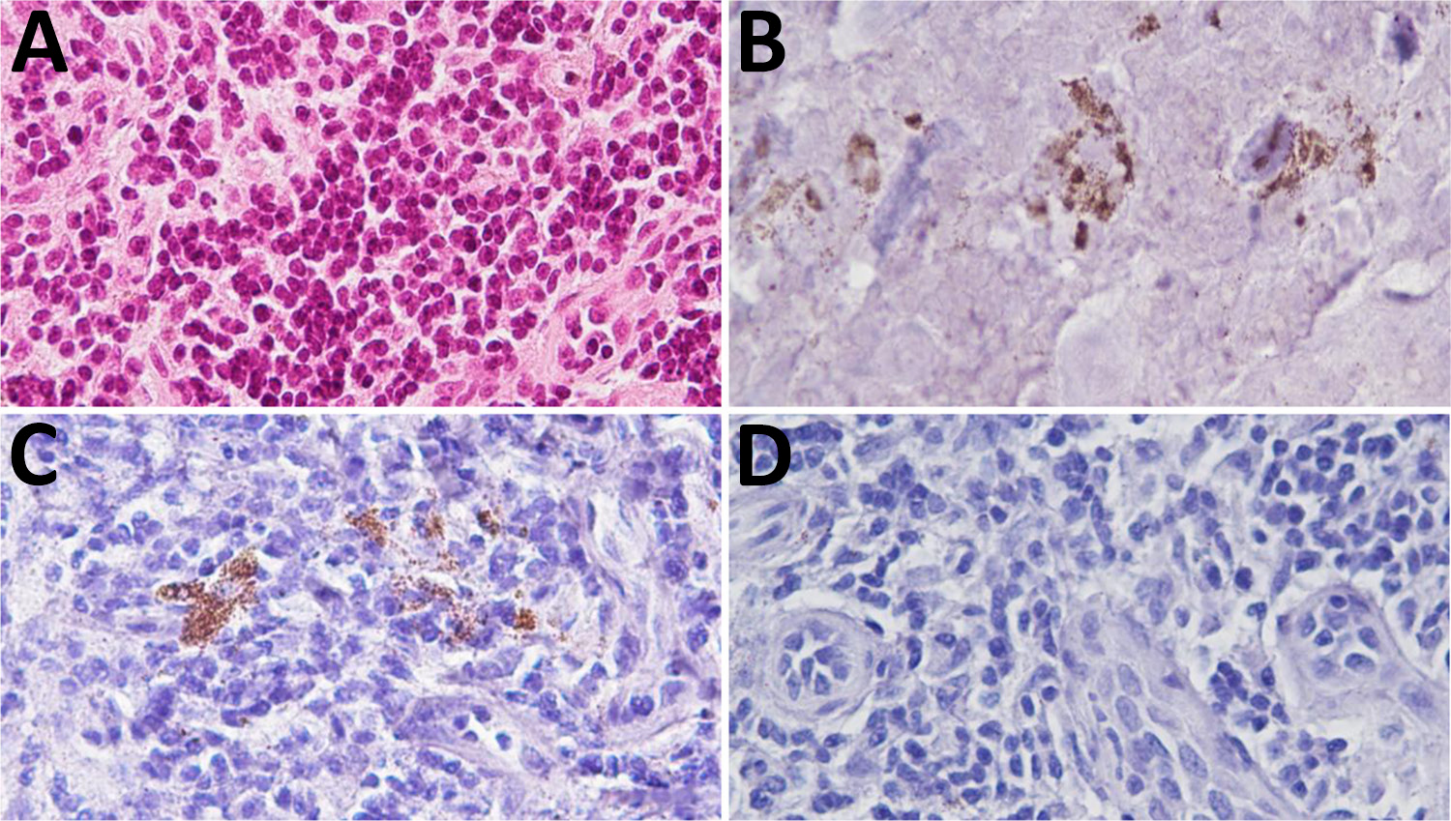
Immunohistochemistry for abnormal disease-associated prion protein (PrP^TSE^) and CD21 on inguinal lymph node of macaque 98CO19 for study of macaques infected with Creutzfeldt-Jakob disease. Adjacent sections were stained with hematoxylin-eosin (A); immunostained for PrP^TSE^ (B) and CD21 (C); and probed without primary antibody as negative control (D). Original magnification ×40.

Brain homogenate from 98CO19 was negative for PrP^TSE^ using multiple biochemical detection methods, and the macaque’s blood was negative by PMCA as well (Table 2). Neuropathologic studies showed no spongiform degeneration or PrP^TSE^ deposits in 98CO19’s brain. However, nasal swab extracts and 2 inguinal lymph node homogenates collected at euthanasia were positive by RT-QuIC (Figure 1) when tested as 1% wt/vol tissue homogenates (4/4 positive wells) but negative in the next 10-fold dilution (0.1% wt/vol), suggesting that very small amounts of PrP^TSE^ were present. PMCA assays of the same 2 tissues were negative. We noted scattered clusters of PrP^TSE^ in sections of the lymph nodes, in the same cells staining for CD21, corresponding to follicular dendritic cells (Figure 2).

We confirmed macaque DEIM to be infected with vCJD by neuropathological and immunohistological examinations of formalin-fixed paraffin-embedded brain tissues and by Western blots of brain suspensions (Appendix Figure, panel A), RT-QuIC of cerebrospinal fluid, and PMCA of blood. PMCA first detected PrP^TSE^ in blood collected at 85 mpt; RT-QuIC showed PrP^TSE^ in nasal swab extracts 7 months later (92 mpt). PrP^TSE^ signals remained positive in both tissues to the end of the study (Appendix Figure). Thus, PrP^TSE^ appeared in those tissues several months before clinical onset, confirming the diagnostic potential of both assays with those materials.

## Conclusion

We report the results of blood transfusions from 3 vCJD-infected macaques into 4 recipient macaques. One macaque developed clinical vCJD 9 years after transfusion, an interval consistent with a survival duration of 6.5–8.3 years seen in human recipients transfused with blood from asymptomatic vCJD-infected donors (*5*). One recipient macaque died early in the experiment, and 2 macaques survived ≥9 years with no clinical signs of vCJD. Macaque 98CO19 revealed PrP^TSE^ signals in nasal swab extracts and inguinal lymph node tissues. Researchers have used lymph node tissue previously to identify macaques incubating vCJD before clinical onset of illness (*11*). Our data from RT-QuIC testing showed reactivity that was weak but unequivocal and reproducible. We confirmed the presence of PrP^TSE^ in lymph node tissue by immunohistochemistry of the same tissue. We concluded that 98CO19 died while infected with vCJD.

Previous researchers exploring TTvCJD transfused to macaques reported a fatal neurologic syndrome, described as a myelopathy, that affected 3 of 7 macaques transfused with blood containing low infectivity (determined by low levels of peripheral PrP^TSE^); the other 4 macaques remained healthy (*12*). The macaques with myelopathy demonstrated clinical signs that included impaired visual acuity and hind limb ataxia, but tests revealed no PrP^TSE^ in brain and other tissues (*12*). Macaque DFOO of our study showed no clinical signs associated with that described myelopathy. Macaque CO1619 died relatively early in the study—too early to know if it had been infected with vCJD or would have developed myelopathy. Our results largely agree with those of Comoy et al., who concluded that not all transfusions transmitted vCJD to macaques (*12*). Similarly, reports from the United Kingdom suggest that not all human recipients surviving 5 years or longer after transfusions developed TTvCJD from a donor with vCJD (*13*). No macaque in our smaller cohort of transfused animals showed evidence of myelopathy.

Over a 10-year period after transfusion, we collected relatively accessible biological materials, such as blood and nasal swab specimens, to test for PrP^TSE^. We first detected PrP^TSE^ in the blood of macaque DEIM 19 months before onset of overt illness, consistent with results of our previous studies (*6*). Nasal swab extracts from the 2 infected macaques became positive for PrP^TSE^ 12 months before signs of illness for DEIM and at euthanasia for 98CO19 (98CO19’s nasal swab specimens were negative 3 months earlier). Those results support potential use of nasal swab specimens as an assay matrix to identify infected persons before clinical onset of vCJD. A caveat is that we do not know how detection of PrP^TSE^ might be affected by prion protein genotype. Research has demonstrated diagnostic accuracy of nasal swab testing for persons with either confirmed or presumed sporadic CJD, but the predictive value of this testing method for detecting cases before onset of neurologic illness remains uncertain (*14*). Acknowledging that our macaques were infected with vCJD, and not sporadic CJD, our results nonetheless suggest that testing nasal swab specimens to detect PrP^TSE^ may be useful in screening persons with family history of CJD, which would enable attempts at early therapeutic intervention (*15*).

Appendix Additional information for abnormal prion protein in nasal swab specimens of macaques infected with Creutzfeldt-Jakob disease.
