# Supplementary material for: Abnormal Prion Protein in Nasal Swab Specimens of Macaques Infected with Creutzfeldt-Jakob Disease
Source: Emerg Infect Dis. 2025 Dec;31(12):2312–5. doi: 10.3201/eid3112.250679 (PMC12782185; doi:10.3201/eid3112.250679)
Supplement: Appendix — Additional information for abnormal prion protein in nasal swab specimens of macaques infected with Creutzfeldt-Jakob disease. [file 25-0679-Techapp-s1.pdf]

Article DOI: http://doi.org/10.3201/eid3112.250679

EID cannot ensure accessibility for supplementary materials supplied by authors. Readers who have difficulty accessing supplementary content should contact the authors for assistance.

Abnormal Prion Protein in Nasal Swab Specimens of Macaques Infected with Creutzfeldt-Jakob Disease

Appendix

Materials and Methods

Experimental animals

Animal studies were carried out in accordance with the recommendations in the Guide for the Care and Use of Laboratory Animals (eighth Ed.) and requirements of U.S. Animal Welfare Regulations. The protocols were reviewed and approved by the Institutional Animal Care and Use Committee at FDA White Oak (ASP2009–14). The FDA White Oak animal program and facilities are fully accredited by AAALAC International. Welfare of caged nonhuman primates was enhanced by a variety of approved methods, including social interaction, toys, novel food items and sensory stimulation (videos). Animals were given enrichment every day. Animals were under constant care of trained technical staff; if an animal required medical attention, a veterinarian determined the final course of action. Animals were monitored twice a day by the animal technical staff and a veterinarian was on call 24 hours a day. 7 days per week. Macaques were euthanized with an IV injection of Euthasol 100 mg/kg as recommended by the American Veterinary Medical Association. All animal procedures were conducted by expert veterinarians assisted by trained veterinary staff.

Macaque transfusions of fresh whole blood and red blood cell-depleted blood

We collected ≈100 mL of whole blood (WB) from donor CO7423 during clinical phase, transferred the blood to a sterile blood bag, and immediately transfused at an approximate flow rate of 10 mL/min into anesthetized recipient CO1619 using an intravenous catheter in the

inguinal vein. A month later, we collected another 100 mL of WB from CO7423 at euthanasia and immediately transfused recipient 98CO19 using the same procedure as above.

We obtained 100 mL of WB from donor CO7422 and C16999 at euthanasia. Those blood samples could not be transfused immediately and so were stored frozen. Prior to storage, we removed red blood cells (red blood cell) by centrifugation of WB at 2000xg for 15 min, collected plasma and buffy coat into separate tubes, and re-centrifuged buffy coat to remove residual red blood cell before storing both components at −80°C until transfusion. On the day of transfusion, we thawed plasma and red blood cell-depleted buffy coat, combined them into a sterile blood bag containing ≈60 ml final volume (red blood cell-depleted whole blood), mixed and transfused into anesthetized recipient macaques DEIM and DFOO as described above. We monitored all transfused macaques during and after transfusion until they were fully recovered.

Transfused macaques were observed daily for signs of disease onset. At euthanasia, we collected ≈150–200 mL of blood, nasal swabs, and cerebrospinal fluid (only DEIM and DFOO) and conducted full necropsy. For each tissue collected, we stored one piece in the freezer for biochemical analysis and one piece in formalin for histology. The brain was further cut into anatomic sections: occipital, frontal, cerebellum, and parietal.

Testing of PrP^{TSE} in macaque tissues and fluids

We collected 30 ml of whole blood and nasal swabs every 3–4 months. We detected PrP^{TSE} in whole blood samples using our PMCA assay (*1*). Briefly, we ultracentrifuged 250 μL of whole blood, resuspended the pellet in 100 μL of 10% normal red-backed vole brain homogenate (substrate) in PMCA buffer and transferred the samples to 0.2 mL tubes containing PTFE beads. We used a titanium cuphorn programmable Misonix Q700 sonicator (QSonica, Newtown, CT, USA). The first round of PMCA was for 72h, the second and third rounds were 24h each. We digested PMCA products with proteinase K (PK) and analyzed the samples by Western blot using 6D11 anti-prion protein antibody (Research Foundation for Mental Hygiene, New York State Institute for Basic Research) and captured the electro-chemiluminescent signals on a Bio-Rad ChemiDoc Imaging System.

We collected olfactory mucosa (nasal swabs) using Puritan PurFlock Ultra Flocked Collection Device (Puritan Diagnostics, Guilford, ME) by gently inserting the brush into macaque nostrils and rotating the brush 10 times. We placed the brush in a tube containing 1 mL

PBS, vortexed, discarded the brush, centrifuged at 10000 g for 15 min, and recovered the pellet. Next, we homogenized the pellets in 200 µL of fresh PBS using glass beads and a tissue homogenizer Beadbeater (Mini Beadbeater, Biospec Products, Bartlesville, OK, USA) for 10 sec. We performed RT-QuIC assays according to our protocol (*2*). Briefly, we used 0.1 mg/mL hamster recombinant PrP purified as previously described (*3*) and RT-QuIC cocktail supplemented with 1x N2 in PBS and 10 µM thioflavin T. We diluted samples in PBS containing 1x N2 and 0.1% SDS and initiated RT-QuIC assay with 2 µL of sample in 98 µL of cocktail (100 µL per well). We tested each sample in quadruplicate wells in a 96-well plate and incubated the plate in a FLUOstar Omega reader (BMG Labtech, Cary, NC) at 50°C for 48 h with shaking in cycles of 1 min on and 1 min off.

We homogenized in a beadbeater brain, spleen, ileum, and lymph nodes diluted in homogenization buffer (10 mmol/L Tris (pH 7.4), 100 mmol/L NaCl, 123 mmol/L sodium deoxycholate, 7.8 mmol/L EDTA and 0.5% wt/vol Igepal CA-630) using 1.0 mm glass beads for a total time of 3 min.

Histology and immunohistochemistry of macaque tissues

We used formalin-fixed, paraffin-embedded tissue sections and published protocols (*4*). We stained brain sections with hematoxylin-eosin (HE) and for immunohistochemical studies we probed sections with anti-PrP Mab (6H4, Prionics, Zurich, Switzerland) and detected astrogliosis using a polyclonal antibody to bovine glial fibrillary acidic protein (GFAP, Dako North America, Carpinteria, CA). We used antibody against CD21 as a marker for follicular dendric cells in macaque lymph node tissue (*5*). It should be noted that lymph node tissue used in this study had been stored frozen. Two independent observers evaluated sections of the brain slices for the presence of spongiform degeneration (vacuolation), PrP^{TSE} accumulation (plaques, grains) and astrogliosis (reactive astrocytes).

References

1. Yakovleva O, Bett C, Pilant T, Asher DM, Gregori L. Abnormal prion protein, infectivity and neurofilament light-chain in blood of macaques with experimental variant Creutzfeldt-Jakob disease. J Gen Virol. 2022;103:001764. PubMed

2. Bett C, Grgac K, Long D, Karfunkle M, Keire DA, Asher DM, et al. A Heparin Purification Process Removes Spiked Transmissible Spongiform Encephalopathy Agent. AAPS J. 2017;19:765–71. PubMed

3. Kaelber N, Bett C, Asher DM, Gregori L. Quaking-induced conversion of prion protein on a thermal mixer accelerates detection in brains infected with transmissible spongiform encephalopathy agents. PLoS One. 2019;14:e0225904. PubMed

4. Piccardo P, Cervenak J, Yakovleva O, Gregori L, Pomeroy K, Cook A, et al. Squirrel monkeys (Saimiri sciureus) infected with the agent of bovine spongiform encephalopathy develop tau pathology. J Comp Pathol. 2012;147:84–93. PubMed

5. Sasaki K, Doh-ura K, Ironside J, Mabbott N, Iwaki T. Clusterin expression in follicular dendritic cells associated with prion protein accumulation. J Pathol. 2006;209:484–91. PubMed

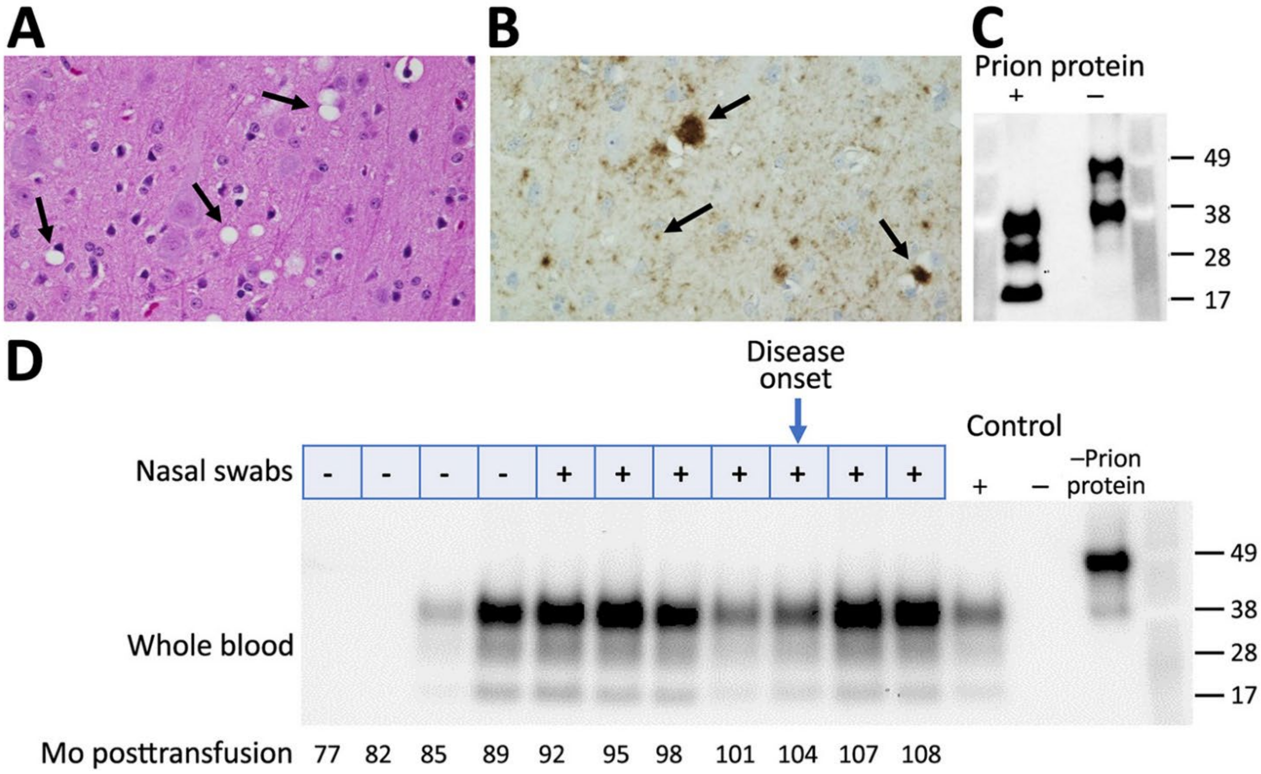

Appendix Figure. Immunohistochemistry for PrP^{TSE} on cerebral cortex of macaque DEIM. Adjacent sections stained with hematoxylin-eosin (A) and immunostained for PrP^{TSE} (B). Presence of vacuolation, PrP^{TSE} plaques and fine punctuated deposits (arrows). No florid plaques were detected. Magnification 40x. (C) PrP^{TSE} signals in DEIM brain homogenate, with and without proteinase K digestion, on Western blot using anti-PrP antibodies. (D) Western blot of PMCA products and reactivity of RT-QuIC with DEIM

samples. Combined results of PMCA with whole blood (lower panel) and RT-QuIC with nasal swab extracts (upper panel). The arrow points to 104 mpt when DEIM first showed clinical onset. Months post-transfusion (mpt) indicated at the bottom of the Western blot reports the time of collection of the samples. Positive control was PMCA product with macaque vCJD brain homogenate and negative control was PMCA product with macaque normal blood. The lane marked “-PK” corresponds to normal prion protein from 0.1% vole brain homogenate without PK treatment. RT-QuIC results are displayed as positive or negative and are aligned with the mpt reported in the figure.
